# Therapeutic effects of garlic (*Allium sativum*) on female reproductive system: A systematic review

**DOI:** 10.1016/j.heliyon.2023.e22555

**Published:** 2023-11-20

**Authors:** Fatemeh Jafari, Somayyeh Khalilzadeh, Fatemeh Nejatbakhsh, Maziar Naderie

**Affiliations:** a Zanjan Applied Pharmacology Research Center, Zanjan University of Medical Sciences, Zanjan, Iran; b Department of Traditional Medicine, School of Medicine, Zanjan University of Medical Sciences, Zanjan, Iran; c Department of Traditional Medicine, School of Persian Medicine, Tehran University of Medical Sciences, Tehran, Iran; d Food Microbiology Research Center, Tehran University of Medical Sciences, Tehran, Iran; e Department of Environmental Health Engineering, School of Public Health, Tehran University of Medical Sciences, Tehran, Iran

**Keywords:** Therapeutic effects, Garlic, Female reproductive system, Persian medicine (PM)

## Abstract

Complementary and alternative medicine, including Persian medicine (PM), offers a variety of disease prevention and treatment methods, including the application of medicinal plants. The health of the reproductive system is an important issue for women, and understanding the potential effect of garlic (*Allium sativum*) for preventive and therapeutic purposes in this field is of interest. This systematic review focused on the effects of garlic on the female reproductive system. The method involved a comprehensive search of relevant literature on experimental animal studies using electronic databases from January 2010 to September 2023, followed by selection of eligible studies and data extraction. A total of 18 studies met the inclusion criteria and were included in the review. This review reported that garlic may have positive effects on women's reproductive health, such as improving hormonal balance, relieving PMS symptoms, and potentially supporting fertility outcomes. This review revealed that garlic compounds such as *allicin* and *ajoene* can modulate various aspects of the female reproductive system, including regulation of the menstrual cycle, hormonal balance, fertility, and reproductive disorders. This review determined that further research is needed to elucidate the molecular pathways and direct effects of garlic on the female reproductive system. Although garlic has many potential health benefits, it should not be used as a substitute for medications.

## Graphical abstract

**Figure:**
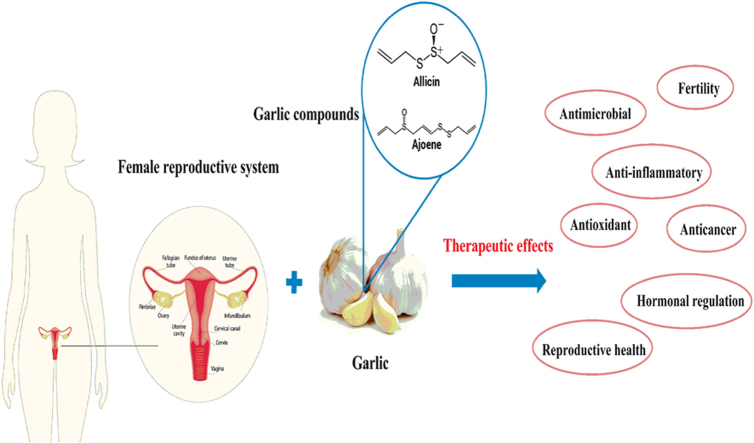

## 1 Introduction

Reproductive health and women's diseases are major challenges in the field of medicine, because these problems affect people's health in different ways and impose a heavy economic burden on societies [1]. Today, there is a great desire to use complementary and alternative medicine in health-related problems [2,3]. Complementary and alternative medicine, including Persian medicine (PM), proposes a variety of treatment methods, including the use of medicinal plants [4,5]. The use of herbal medicines is one of the important complementary medicine solutions for the prevention and treatment of some diseases around the world [6]. In PM, different parts of plants such as roots, stems, leaves and flowers are used as herbal medicines [4,7,8]. These herbal medicines can be used as extracts or decoctions or in medicinal forms such as tablets and ointments [9]. PM is generally based on traditional medicine practices, although some of it is supported by scientific research and clinical trials [10]. PM is commonly used for a wide range of problems, including digestive disorders, respiratory ailments, skin problems, hormonal imbalances, and immune system weakness [11]. Some of the commonly used herbs used in traditional medicine are: ginger, turmeric, ginseng, chamomile and garlic [12]. Garlic (*Allium sativum*) has been used in traditional medicine for centuries because of its many health benefits [13]. It is an edible plant from the *Liliaceae* family. Garlic has been used as a spice to improve the taste of food since ancient times. Garlic has been studied for a long time due to its medicinal effects in the treatment of various human diseases [14]. Studies have shown that garlic has many beneficial properties, including antibacterial, antifungal and antiviral effects. Recently, the potential effects of garlic on the reproductive system of women have been noticed [15]. Garlic has a positive effect on cardiovascular health by reducing blood pressure, reducing cholesterol levels and preventing blood clots [16]. Furthermore, garlic has antimicrobial properties and can strengthen the immune system. This plant has been traditionally used to prevent and treat colds and flu. In addition, garlic contains compounds that have anti-inflammatory effects that reduce inflammation in the body [17]. Some studies have shown that garlic may have anti-cancer properties. This plant inhibits the growth of cancer cells to some extent and may have a preventive effect against all types of stomach, intestine and bladder cancers [18]. Garlic can also improve digestion by stimulating the production of digestive enzymes and the growth of natural intestinal flora. In addition, according to studies, garlic may be effective in reducing the symptoms of digestive disorders such as gastritis and peptic ulcers [19]. Garlic contains antioxidants that protect the body against oxidative stress and free radical damage that contribute to aging and certain diseases [20]. The female reproductive system is a complex set of organs that plays an important role in reproduction. It includes structures such as the ovaries, fallopian tubes, uterus, and vagina that work to facilitate menstruation, ovulation, and pregnancy [21]. Maintaining a healthy reproductive system is very important for mental health and fertility in women. Studies have shown that garlic may have beneficial effects on the female reproductive system [22]. For example, it has been reported that this plant has antimicrobial properties, which can prevent and treat infections related to external reproductive organs [23]. Traditionally, garlic has been used to treat vaginal yeast infections. Its antifungal properties can be effective in preventing and reducing such infections. Due to its anti-inflammatory properties, garlic can also reduce inflammation in the reproductive system and relieve symptoms associated with conditions such as endometriosis and pelvic inflammatory disease [6,24]. Some studies have evaluated the effects of garlic on hormone regulation in women. Hormones play an important role in regulating the menstrual cycle and the normal reproductive process [25]. Studies have reported that garlic may affect certain hormones such as estrogen and progesterone, although more studies are needed to clearly understand these effects [26]. Moreover, some animal studies have shown that garlic may improve fertility, including increasing the number of viable eggs in female animals. However, in order to reach more definitive results, human clinical trials are needed [27]. Garlic contains many compounds including various vitamins B2, B6, B1, A and C, a large amount of antioxidants, flavonoids and phenolic and sulfur compounds [28]. *Allicin* and *ajoene* are two biologically active compounds containing garlic sulfur [29]. *Allicin* (dialkyl thiosulfinate) plays a key role in the medicinal properties of garlic and is formed by the action of allinase on alliin (S-alkyl-l-cysteine sulfoxide) [30,31]. *Allicin* and *ajoene* have been noted for their potential therapeutic effects. It is reported that these compounds have antimicrobial, anti-inflammatory and antioxidant properties. However, limited research has been conducted on their effects on the female reproductive system [32]. The effects of garlic on hormonal balance, fertility and sexual health have been studied. In a study, Jiao et al. investigated the use of herbal medicines used to treat menstruation in women and the prevalence of menstrual diseases in different regions [12]. Moreover, Raji et al. evaluated the effects of aqueous garlic extract on some aspects of reproduction in female albino rats (Wistar strain). Their results showed that the aqueous extract of garlic has no harmful effect on the reproductive performance of female rats [33]. In another study, Wassem et al. carried out a hormonal study on the efficacy of garlic extract in preventing fallopian tube lead acetate toxicity [34]. Similarly, Bashir et al. conducted a study on the effect of aqueous garlic extract on the changes caused by androgens in the ovaries of female rats before puberty. The results of their study showed that aqueous extract of garlic reduced the number and size of cystic follicles in androgen-treated ovaries of immature rats [35]. In addition, Musavi et al. reported that the increase in fertility of garlic is probably due to its antioxidant properties [29]. Most of the researches in this field are preliminary and have been done on animal models on a laboratory scale. More research is needed to better understand the effects of *allicin* and *ajoene* on the female reproductive system and their potential therapeutic applications. In addition, individual responses to these compounds may vary and there may be side effects or drug interactions. Garlic is a well-known medicinal plant with food and medicinal uses that has been used in different cultures for years to prevent and treat diseases [4]. Since the studies conducted in the field of PM have recommended the use of garlic for the prevention and treatment of various diseases, this review can be useful for researchers. In this study, the medicinal effects of garlic on diseases related to the female reproductive system were investigated and interpreted.

## 2 Methodology

The purpose of this systematic review was to synthesize existing animal studies on the therapeutic effects of garlic on diseases related to the female reproductive system. A systematic search of electronic databases was conducted to identify relevant studies that investigated the effects of garlic compounds on diseases of the female reproductive system. The key questions in the systematic review on the therapeutic effects of garlic on the female reproductive system have been demonstrated in Table 1. These questions helped guide the systematic review process by providing a clear focus on specific aspects of garlic's therapeutic effects on gynecological diseases. In light of these questions, the aim of this review was to provide a comprehensive synthesis of the available evidence and help understand the potential benefits of garlic in this area.

**Table 1:** The questions of this research.

No.	Question
1	Which reproductive problem is most commonly treated with garlic?
2	What are the potential therapeutic effects of garlic on the female reproductive system?
3	Does consuming garlic have a positive effect on health and menstrual regulation?
4	Can garlic be used as a natural remedy for common female diseases or disorders?
5	Is there a phytochemical or special compound in garlic that contributes to its therapeutic effects on the female reproductive system?
6	What is the optimal dose and duration of garlic consumption for optimal therapeutic results?
7	Are there any potential side effects or contraindications of garlic in relation to the female reproductive system?
8	What is the overall quality and strength of the available evidence regarding the therapeutic effects of garlic on the female reproductive system?
9	Are there gaps in the current research that warrant further investigation?

### 2.1 Study protocol

A protocol was designed to describe the objectives, research questions, and methods used in the systematic review. It defined the methodology, study design, search strategy, inclusion and exclusion criteria, data extraction process, quality assessment, data synthesis, interpretation of results, and reporting. In this protocol, the review was done in a precise and transparent manner [36].

### 2.2 Study design

In this study, a qualitative systematic review plan was used, which included the search, selection and systematic analysis of qualitative studies and systematic reviews related to the research topic. These studies were chosen because they investigated the effects of garlic on the function and diseases of the female reproductive system in a laboratory scale and in the form of animal studies. This review followed the guidelines outlined by the Preferred Reporting Items for Systematic Reviews and Meta-Analyses (PRISMA) [36].

### 2.3 Search strategy

The search terms used were “garlic,” “female reproductive system,” “menstruation,” “fertility,” “hormone regulation,” “ovulation,” and “endometrium”. The studies that met specific inclusion criteria were selected for analysis. The review included studies published in English from various databases, such as PubMed, EMBASE, CINAHL, and Scopus from 2010 to 2023. Boolean operators (AND, OR) were also used to enhance search efficacy [36]. The search strategy in the databases has been showed in Table 2.

**Table 2:** Search strategy in the considered databases.

Database	Search strategy	Number of articles retrieved
PubMed	1. (“Garlic” OR “*Allium sativum*”) AND (“Female reproductive system” OR “Women's health” OR “Gynecological disorders”) 2. (“Garlic” OR “*Allium sativum*”) AND (“Menstrual disorders” OR “Dysmenorrhea” OR “PCOS” OR “Endometriosis”) 3. (“Garlic” OR “*Allium sativum*”) AND (“Menopause” OR “Hormonal imbalance” OR “Estrogen” OR “Progesterone”) 4. (“Garlic” OR “*Allium sativum*”) AND (“Uterine health” OR “Vaginal health” OR “Cervical health” OR “Ovarian health”)	976
EMBASE	1. (garlic OR “*Allium sativum*”) AND (female reproductive system OR “Women's health” OR “Gynecological disorders”) 2. (garlic OR “*Allium sativum*”) AND (menstrual disorders OR dysmenorrhea OR PCOS OR endometriosis) 3. (garlic OR “*Allium sativum*”) AND (menopause OR “Hormonal imbalance” OR estrogen OR progesterone) 4. (garlic OR “*Allium sativum*”) AND (uterine health OR vaginal health OR cervical health OR ovarian health)	843
CINAHL	1. (garlic OR “*Allium sativum*”) AND (female reproductive system OR “Women's health” OR “Gynecological disorders”) 2. (garlic OR “*Allium sativum*”) AND (menstrual disorders OR dysmenorrhea OR PCOS OR endometriosis) 3. (garlic OR “*Allium sativum*”) AND (menopause OR “Hormonal imbalance” OR estrogen OR progesterone) 4. (garlic OR “*Allium sativum*”) AND (uterine health OR vaginal health OR cervical health OR ovarian health)	720
Scopus	1. (garlic OR “*Allium sativum*”) AND (female reproductive system OR “Women's health” OR “Gynecological disorders”) 2. (garlic OR “*Allium sativum*”) AND (menstrual disorders OR dysmenorrhea OR PCOS OR endometriosis) 3. (garlic OR “*Allium sativum*”) AND (menopause OR “Hormonal imbalance” OR estrogen OR progesterone) 4. (garlic OR “*Allium sativum*”) AND (uterine health OR vaginal health OR cervical health OR ovarian health)	213

### 2.4 Inclusion and exclusion criteria

The inclusion and exclusion criteria for the qualitative systematic review on the effects of garlic on the female reproductive system have been described in Table 3.

**Table 3:** Inclusion and exclusion criteria.

Inclusion Criteria	Exclusion Criteria
**Animal studies**	Other studies
**Studies published in English and Persian**	Studies published in other languages
**Studies conducted on female participants.**	Studies conducted on male participants.
**Review studies published from 2010 to 2023**	Review studies published up to 2010
**Studies investigating the effects of garlic on the female reproductive system**	Studies investigating the effects of garlic on the other system
**Studies published in peer-reviewed journals**	Studies published in non-peer-reviewed sources
**Studies conducted on animals**	Studies conducted on humans

### 2.5 Data extraction

The information of the articles was reviewed independently by two authors based on the inclusion and exclusion criteria. Finally, both authors classified the data, and in cases where the data were inconsistent, a third author's comments were used. The information obtained from the articles was entered into the checklist according to the quality approval of the articles. The checklist included: name of the author(s), year of publication, study design, sample size, intervention details, results and conclusions.

### 2.6 Quality assessment

The quality assessment of the included studies was assessed using the SYRCLE risk of bias tool. This tool is designed for animal studies and is the best quality assessment tool in this field. SYRCLE tool consists of ten areas including selection bias, performance bias, diagnosis bias, attrition bias, reporting bias and other sources of bias. This tool is an advanced and improved model of previous quality assessment tools that are specific to animal and preclinical studies, and some of the shortcomings of previous tools related to clinical trials have been corrected [37].

### 2.7 Data synthesis

In this study, combined data from included studies were analyzed using thematic analysis. As well as, common themes and patterns related to the effects of garlic on the performance and diseases of the reproductive system of women were identified. The findings were then interpreted to generate meaningful insights and recommendations.

### 2.8 Interpretation of results

The interpretation of the results included the analysis of the themes identified in relation to the research questions and objectives. The results obtained in the included studies related to the effects of garlic on the reproductive system of women were reviewed. Additionally, final conclusions for health care providers, policy makers, and future research are discussed.

### 2.9 Reporting

The results of the qualitative systematic review were reported following the guidelines of the PRISMA statement (Appendix 1, Appendix 2). Recommendations for practice and future research were also included.

## 3 Results and discussion

**Fig. 1:**
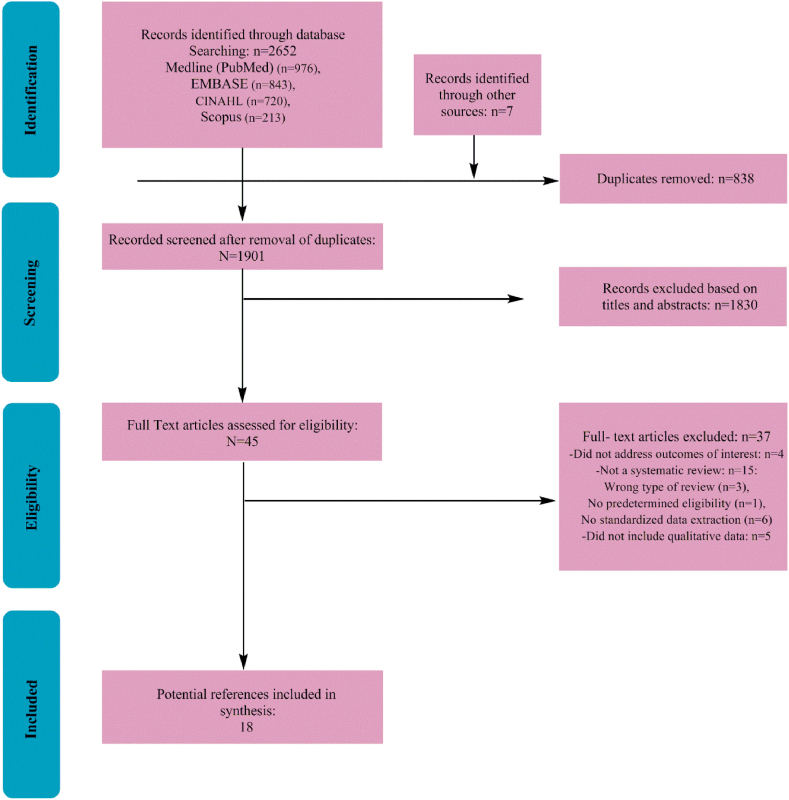
Trend of screening and choosing articles based on PRISMA guidelines.

A systematic review on the effects of garlic on the female reproductive system was conducted to evaluate the scientific evidence in the available studies. This review collected and analyzed various studies focusing on the effects of garlic compounds on the performance, health and diseases of the female reproductive system. The results of this systematic review showed that garlic compounds have significant effects on the reproductive system of women. A total of 18 studies met the inclusion criteria and were included in this review (Fig. 1). The characteristics of the studies included in this review are described in Table 4. This review showed that garlic is generally known for its medicinal properties, including anti-inflammatory, antioxidant and antimicrobial properties. Based on the reviewed studies, there is limited scientific evidence about the effects of garlic on the female reproductive system. Garlic is traditionally used as a natural remedy for various women's ailments. The results of this review study showed that garlic has potentially beneficial effects on hormonal regulation, enhancing fertility, regulating the menstrual cycle and reducing inflammation in the female reproductive system [38]. Some studies have reported that garlic may have beneficial effects on the female reproductive system by reducing menstrual pain and regulating menstrual cycles. However, more research is needed to find out the exact mechanisms. Additionally, some studies have shown the potential benefits of garlic compounds on women's reproductive health. Garlic can help neutralize free radicals because it has no side effects and also contains flavonoids, vitamins, and fructose and sulfur compounds [39]. Several bioactive compounds in garlic, including *allicin* and diallyl sulfide, are thought to be responsible for promoting reproductive health. These compounds may modulate hormone levels and have anti-inflammatory and antioxidant activities. As a result, they affect various aspects of the female reproductive system. When fresh garlic is crushed, alliin is converted to *allicin* by allinase [40]. The produced allicin is unstable and rapidly hydrolyzes to other sulfur-containing compounds such as diallyl disulfide, diallyl trisulfide (DAT), and diallyl tetrasulfide. Diallyl trisulfide has the highest abundance (45 %) among the organic sulfur compounds found in garlic and has potential medicinal functions [41]. This review showed that most of the reviewed studies were at high risk of bias (Table 5). In most of these studies, randomization in sampling and research blinding were not clearly mentioned. Among the 10 reviewed studies, garlic extract was given to animals in the form of raw plant and powder. In these studies, the maximum and minimum sample sizes were 62 and 10 mice, which were investigated in experimental studies [42]. The control group received distilled water, normal saline, and powder containing starch, and the treatment group received garlic extract, adriamycin, titanium dioxide, garlic oil, N-acetylsicine, vitamin E, cadmium, cooked garlic, and garlic juice [29].

**Table 4:** Characteristics of included studies in the review research.

First author and reference	Title	Study type	Purpose/aim/objectives of the study	Design and study sample	Investigation methods	Major findings	Year
Falahatian et al. [25]	Modulatory effects of R10 fraction of garlic (*Allium sativum* L.) on hormonal levels, T cell polarization, and fertility-related genes in mice model of polycystic ovarian syndrome	Animal experimental study	To evaluate the potential immune-modulatory effects of R10 fraction of garlic in a mouse model of PCOS.	The therapeutic effects of R10 fraction were investigated in the PCOS mouse model. To do this, PCOS was induced by intramuscular injection of estradiol valerate.	Treatment with R10 fraction, isolated from garlic, and changes in hormone levels (estradiol, progesterone, and testosterone), markers of T cell polarization (IFN-γ, IL-4, and IL-17), and expression of fertility-genes (Gpx3 and Ptx3) were investigated.	Hormonal levels were increased in the PCOS model compared to normal animals, but were significantly modulated after treatment with R10 fraction. Treatment with R10 fraction also showed modulating effects on T cell polarization by increasing IL-4 and decreasing IL-17 and IFN-γ levels. Our study clarified that the R10 fraction of garlic has immuno-modulatory effects in reducing PCOS symptoms.	2022
Ghyasi et al. [43]	Combination Effect of Voluntary Exercise and Garlic (*Allium sativum*) on Oxidative Stress Biomarkers and Lipid Profile in Healthy Rats	Animal experimental study	To investigate the combination effect of voluntary exercise and garlic on serum oxidative stress biomarkers and lipid profile in healthy rats.	The rats were randomly assigned to four groups (n = 7): Control, Garlic, Exercise, and Garlic with Exercise.	Mice were fed fresh raw garlic homogenate by oral gavage (250 mg/kg) or subjected to voluntary exercise using stainless steel wheels alone or together for 6 weeks.	The serum levels of glutathione peroxidase (Gpx), superoxide dismutase (SOD), catalase (CAT) and total antioxidant (TAC) increased significantly after the aforementioned interventions. The results showed that simultaneous treatment of mice with garlic and voluntary exercise improved the antioxidant defense system and lipid profile in healthy mice.	2019
Desai et al. [44]	Revealing the Therapeutic Uses of Garlic (*Allium sativum*) and Its Potential for Drug Discovery	Animal experimental study	To investigate the testicular toxicity of the pyrethroid insecticide, deltamethrin (DM) in male albino Swiss mice, *Mus musculus* and to evaluate the protective role of *Allium sativum* (AS) in mitigating the harmful effect of DM.	Forty male mice were divided into five experimental groups (8 mice per every group).	Animals were randomized into control and treated groups and were caged separately. Moreover, the experiments were conducted for duration of 45 days.	The group treated with DM showed a significant increase in the concentration of testicular malondialdehyde (MDA) and cholesterol. In contrast, treatment with Allium satium ameliorated DM-induced oxidative damage and other evaluated indices of testes. DM also showed significant harmful effects on male rats and co-administration of *Allium sativum* ameliorates the harmful effects of DM.	2015
Elkelawy et al. [45]	Effect of garlic (*Allium sativum*) on hematological, biochemical, hormonal and fertility parameters of female Bouscat rabbits	Animal experimental study	To evaluate the effect of garlic on hematological, biochemical, hormonal and fertility parameters of female Bouscat rabbits	This experimental study lasted two months (from 6 to 8 months) to investigate the effects of garlic treatment on blood, biochemical, hormonal and fertility parameters of male Bouscat rabbits. For this purpose, 24 male Bouscat rabbits weighing 3150–3300 g were divided into four experimental groups with equal numbers (6 people).	The first group was used as a control and was injected subcutaneously with saline solution (1 ml of 0.9 % sodium chloride), the second, third and fourth groups were given garlic once a week for 8 weeks with doses of 3, 9 and 27 mg/kg of body were injected subcutaneously. Weight respectively	Fat, total cholesterol and triglyceride levels in male rabbits treated with different doses of garlic decreased with increasing doses of garlic. This reduction was statistically significant with higher doses (9 and 27 mg/kg body weight). Garlic treatment of buck rabbits caused a slight increase in high-density lipoprotein (HDL) levels, while a significant decrease in low-density lipoprotein (LDL) and very-low-density lipoprotein (VLDL) levels. A low dose of garlic improved fertility parameters of buck rabbits.	2020
Hajiuon et al. [46]	Effects of Garlic (*Allium sativum* L.) Hydroalcoholic Extract on Estrogen, Progesterone and Testosterone Levels in Rats Exposed to Cell Phone Radiation	Animal experimental study	Investigating the possible effects of radiation and garlic consumption on estrogen, progesterone and testosterone levels.	The 5 groups of male and 5 female rats were used: control, sham (exposed to light), experiment 1 (receiving garlic extract), experiments 2 and 3 (receiving extract and microwave). After one month, mice were weighed and serum levels of hormones were measured.	Animals were randomly divided into 5 groups of 6 males and 5 females of 6 including control (untreated), sham group (exposed to 900 MHz wavelength), experimental group 1 (received 400 mg/kg of garlic extract), experimental group 2 were placed receiving 200 mg of the extract plus 900 MHz waves, and experimental group 3 (receiving 400 mg/kg of the extract plus 900 MHz waves).	Estrogen decreased and progesterone increased in all groups. Microwaves and garlic extract had fewer effects on women's reproductive system, which is only reflected in serum progesterone concentration. They were also reflected in the number of Leydig cells and serum concentrations of testosterone and estrogen.	2014
Jafari et al. [47]	Comparative effects of garlic (*Allium sativum*) powder and atorvastatin in female reproductive system of hypercholesterolemic rats: A histological and biochemical evaluation	Animal experimental study	Investigating the comparative effect of garlic powder and atorvastatin on reproductive failure caused by hypercholesterolemia in female rats.	48 adult female Wistar rats were divided into eight groups (n = 6) including control, atorvastatin (10 mg/kg per day; oral), atorvastatin (20 mg/kg per day; oral), garlic. powder (100 mg/kg/d; oral), hypercholesterolemia (1.5 mg/kg/d cholesterol; oral), hypercholesterolemia + atorvastatin (10 mg/kg/d), hypercholesterolemia + atorvastatin (20 mg/kg/d) and hypercholesterolemia + garlic powder	After 30 days, mice were euthanized and blood samples were taken from their hearts for serological evaluation. The right ovary was transferred to 10 % formalin for histological analysis, and the left ovary was transferred to −80 °C freezer to evaluate oxidative stress markers.	The number of healthy primary, primary, secondary and antral follicles, catalase activity, total antioxidant capacity (TAOC) as well as estrogen and progesterone levels was lower in hypercholesterolemic rats than in the control group. In addition, the number of primary, secondary and antral atretic follicles and malondialdehyde (MDA) levels were higher in hypercholesterolemic rats. But garlic powder and atorvastatin 10 improved the changes in the mentioned parameters (P = 0.99). Garlic powder improved ovarian toxicity in hypercholesterolemic rats better than atorvastatin.	2021
Risikat et al. [48]	Comparative oestrogenic effects of *Allium sativum* and *Allium cepa* in ovariectomized rats	Animal experimental study	Evaluation of therapeutic effectiveness of *Allium cytium* and *Allium cepa* on estrogenic activities of ovariectomized adult Wistar rats.	Institutional Animal Care and Use Committee (IACUC) guidelines were strictly followed during animal transport (IACUC, 2011). Accordingly, this research was conducted in line with the guidelines of the ethical review committee of the University of Ilorin.	A total of 36 (thirty-six) adult female Wistar rats were fed pelleted feed (breeders mesh) purchased from Ogo-Oluwa Feed, Sango Ilorin and had free access to clean water.	*Allium cepa* and quercetin increased the thickness of the endometrium, increased the number of neurons in cells stained with synaptophysin of the hippocampus and dentate gyrus. *Allium cepa*, which has similar estrogenic properties to the estradiol group in these ovariectomized rats, could provide some ameliorating effects of estrogen deficiency.	2022
Raji et al. [33]	The Effects of Aqueous Extract of *Allium sativum* (Garlic) on Some Aspects of Reproduction in the Female Albino Rat (Wistar Strain)	Animal experimental study	Investigating the effect of its extract on some aspects of reproduction in female albino rats (Wistar strain).	Seventy-five female albino rats in proestrus were divided into five groups of fifteen. Group A was given distilled water as a control, and groups B, C, D, and E were given daily oral doses of 200, 400, 600, and 800 mg/kg body weight of aqueous garlic extract for twenty-eight days and during the period 28–30 days of pregnancy were given.	Five mice in each group were sacrificed on the 14th day and the 28th day for gross and histological examinations in the first stage. The remaining five mice (in each group) were mated in the second step.	The body weight of the uterus and cervix in group E (P < 0.05) has a significant decrease compared to groups A, B, C and D, while the ovaries did not show significant changes. A significant increase in the length of the right uterine horn and a decrease in the length of the left uterine horn (P < 0.05) were observed in groups B, C, D and E compared to group A. Mated mice all became pregnant without abortion. There was no significant difference (p > 0.05) in infant size, live birth weight, and infant mortality among the groups. These findings show that the aqueous extract of garlic has no harmful effect on the reproductive performance of female mice.	2012
Batool et al. [49]	Curative Potentials of Garlic (*Allium sativum*) Extract against Di-(2-Ethylhexyl) Phthalate Induced Reproductive Toxicity in Female Mice	Animal experimental study	To explore the therapeutic potential of garlic (*Allium sativum*) against the toxicity caused by di-(2-ethylhexyl) phthalate (DEHP) in the reproductive system of female rats.	Forty female rats were divided into four groups (n = 10), which (a) control group was given normal food and drinking water, (b) group treated with aqueous extract of garlic (500 mg/kg), (c) group received DEHP 500 mg/kg in corn oil and (d) DEHP + garlic aqueous extract each at a dose of 500 mg/kg body weight.	After completion of the experiment, all animals were dissected through cervical dislocation to obtain reproductive organs. The collected organs were weighed and processed through the conventional histological technique of staining with eosin and hematoxylin.	Treatment with DEHP plus garlic extract showed protective effects on the uterus, such as a significant increase in uterine diameter, muscularity, mean number of endometrial glands, and endometrial epithelial height compared to the DEHP-only group. Hence, garlic extract showed significant amelioration potential against DEHP-induced reproductive abnormalities in female rats.	2022
Iram et al. [50]	Effect of aqueous garlic (*Allium sativum*) extract against di-(2-ethylhexyl) phthalate induced reproductive toxicity in male mice	Animal experimental study	Evaluation of testicular and male reproductive system histopathologies and lipid profile against exposure to diethylhexyl phthalate (DEHP) in mice and the therapeutic potential of aqueous extract of garlic (*Allium sativum*).	Four groups (n = 10) were named and treated (A) control (C): (normal food and drinking water + 0.2 ml corn oil). (b) Aqueous extract of garlic (AGE) group: (500 mg/kg body weight of aqueous extract of garlic). c) DEHP group: (500 mg/kg body weight DEHP, dissolved in corn oil; d) AGE + DEHP group (500 mg/kg body weight garlic aqueous extract, and DEHP 500 mg/kg body weight corn oil dissolved).	Doses were given once daily by gavage for 28 days, and on day 29, all animals were killed by cervical dislocation, and reproductive organs and blood samples were collected.	Exposure to DEHP on body weight, testis weight, serum cholesterol, triglyceride, lipid profile, mean seminiferous tubule cross-sectional area, seminiferous tubule lumen ACSA, spermatogenic cells, Leydig cell count, diameter of vas deferens, lumen, Muscle thickness and height of epithelial cells of the vas deferens. This study found that exposure to DEHP can be harmful to male reproductive health, and that an aqueous extract of garlic can reduce the toxic effects of DEHP in male rats.	2022
Hagag et al. [51]	Effect of Feeding Pomegranate (Punica granatum) Peel and Garlic (*Allium sativum*) on Antioxidant Status and Reproductive Efficiency of Female Rabbits	Animal experimental study	To investigate how adding pomegranate peel (PP), garlic powder (GP) or a mixture of the two to their diet affects their weight, number of offspring, reproductive performance, blood and antioxidant indices, as well as liver and kidney functions.	A total of 20 female and adult mixed rabbits aged 4.5–5 months with an average weight of 3.05 ± 0.63 kg were divided into four experimental groups (5 people).	The first group was fed with basal diet and was considered as control animals, while the second, third and fourth groups were fed with basal diet supplemented with PP 3.0 %, GP 3.0 % and a mixture of PP 1.5 % + GP 1.5. %, Respectively. After 2 weeks of feeding the experimental diets, natural mating was performed with untreated pairs.	The results showed that creatinine levels in PP (3 %) and GP (3 %) rabbits decreased significantly compared to control rabbits. The results of superoxide dismutase, catalase, glutathione and total antioxidant capacity also indicated a significant decrease in the groups treated with GP (3 %) compared to other treated groups. In conclusion, pomegranate is a promising ingredient to include in the diet of rabbits, followed by garlic to increase reproductive efficiency.	2023
Parvez et al. [52]	Antifertility Activity of Methanol Bulb Extract of *Allium sativum* on Swiss Albino Male Mice and Teratogenic Effect on Neonates of female Mice	Animal experimental study	Investigating the anti-fertility activity of methanolic onion extract of *Allium satium* on Swiss albino male rats.	Adult albino Swiss mice (number = 22) aged 30–35 days were used. Mice were collected from Faculty of Pharmacy, Jahangirnagar University. Mice weighing about 30–40 g were placed in colony cages (4 mice per cage). Albino Swiss mice were divided into three groups.	The plant of *Allium satium* L. was cut into small pieces and 500 g of chopped onion of *Allium satium* was soaked in 1000 ml of methanol and placed in a rotary shaker with continuous shaking for 7 days.	The results of this study showed that the methanolic onion extract of *Allium sativum* plant has an anti-fertility effect on male mice and has no teratogenic or non-beneficial effect on female mice.	2015
Kadir et al. [53]	Oestrogenic Effects of Onion and Garlic Extracts: Potential Alternatives to Synthetic Oestradiol?	Animal experimental study	Evaluating the estrogenic activities of onion and garlic and their effects on the uterus of adult female Wistar rats.	A total number of 30 rats including five rats in six groups were used. Group I received only feed and water and served as control. The second group (estradiol group) was administered oral estradiol 10 μg/kg. Groups III and IV were given 1.14 g/kg and 1.7 g/kg body weight of onion extract respectively, while groups V and VI were given 1.14 g/kg and 1.7 g/kg body weight of garlic extract respectively for four weeks.	The microscopic structure of the uterus of all studied animals was observed under a light microscope after the preparation of tissues (uterus) and subsequent staining with hematoxylin and eosin dyes. And serum estrogen levels were measured and compared after menopausal women and women of all ages with four weeks of treatment.	Animals in the control group had higher estrogen levels than other treatment groups, including the estradiol group. The results of this study also showed that the use of onion or garlic as a potential substitute for synthetic estradiol in the treatment of estrogen deficiency associated with menopause cannot be proven.	2018
Ene et al. [54]	The effect of *Allium Sativum* (Garlic) on Pregnancy, Fetal Weights, and Some Hematological Parameters in Albino Rats	Animal experimental study	Investigating the effect of *Allium satium* (garlic) on pregnancy, fetal weight and some blood parameters in Wistar rats	The animals were divided into four (4) groups A, B, C and D of ten female mice (n = 10). Group A (control) was fed with normal rat food and water ad libitum. Group B induced pregnancy and treated with aqueous extract of AS, group C (n = 10) pregnant and receiving AS extract, group D (n = 10) non-pregnant and receiving AS extract.	Each of the basic parameters of the mice was determined before the induction of pregnancy and then the parameters were evaluated weekly.	There was a significant difference between groups B, C and D compared to group A on days 14 and 21. The number of platelets decreased significantly in all groups and no significant difference was observed between groups compared to A. The results showed that the consumption of garlic during pregnancy has a beneficial effect in reducing the weight of the mother/fetus.	2017
Sheweita et al. [55]	Antioxidants (selenium and garlic) alleviated the adverse effects of tramadol on the reproductive system and oxidative stress markers in male rabbits	Animal experimental study	Investigating the protective effects of antioxidants (garlic and selenium) against the toxic effects of tramadol on the characteristics of semen, steroid hormones, protein expression of different cytochrome P450 isozymes and the activity of antioxidant enzymes in the testes of rabbits.	Western immunoblotting, spectrophotometry and histology methods were used in this study.	Tramadol (1.5 mg/kg body weight) orally to male rabbits for up to three months (three times a week) and after treatment with garlic (800 mg/kg body weight) and/or selenium (1 mg/kg body weight) of rabbits was administered.	The results showed that such toxic effects of tramadol were reduced and returned to their normal level after pretreatment of rabbits with garlic, selenium and/or their combination. This finding may pave the way for a new approach to reduce tramadol toxicity.	2022
Bashir et al. [35]	Effect of aqueous garlic extract on androgen induced changes in ovaries of prepubertal female albino rats	Animal experimental study	Evaluation of the effect of garlic extract on androgen -induced changes in the ovary of prenatal rats and assessment of protection provided by aqueous garlic extract.	Fifty pre -operative rats at the age of 21 were divided into five groups A, B, C, D and E. Group A as a witness and received 5 ml/kg per day from subcutaneous propylene glycol for 14 days. Group B Testosterone Propionate (TP) dissolved 10 mg/kg/day at 5 ml/kg subcutaneous propylene glycol for 14 days.	Group C received 10 mg/kg per day at 5 ml/kg of subcutaneous propylene glycol and the age of 200 mg/kg orally for 14 days. Group D was dissolved for 14 mg/kg per day at 5 mg/kg of propylene glycol for 14 days and water garlic extract 200 mg/kg orally from day 14–21. Group E 10 mg/kg dissolved In 5 ml/kg of propylene glycol for 14 days without intervention by day 21. Group A, B and C animals were sacrificed on day 15 and Group D and E on day 22, the ovaries were removed and examined.	Histological sections increased significantly in the number of large cystic and anthral follicles in groups B and C, respectively. However, the number and size of cystic follicles decreased after treatment with water extract. The results showed that the extract of water garlic prevents and reduced the number and size of cystic follicles in ovaries treated with androgen of immature mice.	2017
Ukpanukpong et al. [56]	Hormonal and electrolyte assessment on the effect of garlic (*Allium sativum*), Vitamin C and E in tramadol induced toxicity in female Wistar rats	Animal experimental study	Evaluation of the antioxidant effect of vitamin C, E and garlic on toxicity caused by tramadol in wistar rats	The rat (35) was dedicated to five studies from seven mice. Group A: NT Determined Mice contains positive control mice and without any therapy B: TM designated mice with 0.2 mg of tramadol C tramadol: specified TMVC includes 0.2 mg tramadol mice and 0.2 ml vitamin C D: The specified TMVE included mice prescribed 0.2 mg tramadol and 0.2 ml vitamin E. Group E: TMG included mice with 0.2 mg tramadol and 0.2 ml garlic	It was adapted to the animals for two weeks and their weight was measured before treatment. At the end of the experimental period, the mouse took a day overnight and was sacrificed to anesthesia with the cervical displacement. After the mice were sacrificed, 2–4 ml of blood was collected from each mice and placed in specific sterile bottles for hormonal and electrolyte analysis.	The total level of vitamin C, E and garlic antioxidants increased significantly compared to the negative control group. In TmVC, TmV, TmG groups, the amount of bicarbonate increased significantly compared to the control group. The levels of serum hormones of the LH group and estrogen showed a significant decrease in the negative control group compared to the positive control group. But in groups of TmVC, TmV and TmG, the LH and estrogen group increased significantly.	2019
Al-Shaibani et al. [57]	Histological Study of Aqueous Extracts Leek *Allium porrum* L. in Female Reproductive System of Laboratory White Rats	Animal experimental study	To evaluate the impact of the water extracts of the *porrum* leaf of alum on the reproductive system in the female albino mice.	The study included 30 female white rats type **Rattus rattus** age average between 8 and 12 weeks.	The animals were placed in appropriate laboratory conditions, temperature of about 30–20° and at a constant speed of lighting system 13 h and 11 h of darkness.	Aqueous Extracts Leek *Allium porrum* had anti -fertility effects on white mice through negative effects on the ovaries and the uterus. It also affected the number of follicles and the thickness of the endometrium and mythometer.	2014

**Table 5:** Risk of bias for animal studies, using the SYRCLE risk of bias tool.

	1	2	3	4	5	6	7	8	9	10
Study	**Selection bias1**	**Selection bias2**	**Selection bias3**	**Performance Bias1**	**Performance Bias2**	**Detection Bias1**	**Detection Bias2**	**Attrition bias**	**Reporting bias**	Other potential bias
Falahatian et al.	×	✓	✓	?	×	?	×	?	✓	✓
Ghyasi et al.	✓	✓	✓	?	×	×	؟	✓	✓	✓
Desai et al.	✓	✓	✓	?	?	?	?	✓	✓	✓
Elkelawy et al.	✓	✓	✓	?	?	×	؟	✓	✓	✓
Hajiuon et al.	✓	✓	×	?	×	؟	×	؟	✓	✓
Jafari et al.	؟	✓	؟	?	?	؟	؟	؟	✓	؟
Risikat et al.	✓	✓	؟	?	×	؟	؟	؟	✓	✓
Raji et al.	✓	✓	✓	?	?	؟	؟	×	✓	✓
Batool et al.	✓	؟	✓	?	?	✓	؟	؟	؟	؟
Iram et al.	✓	✓	✓	✓	?	?	?	?	✓	✓
Hagag et al.	✓	✓	✓	?	?	?	?	?	✓	✓
Parvez et al.	✓	✓	؟	?	?	؟	؟	✓	✓	✓
Kadir et al.	✓	✓	؟	?	?	✓	؟	؟	✓	؟
Ene et al.	✓	✓	✓	?	?	؟	؟	؟	✓	✓
Sheweita et al.	✓	✓	؟	?	✓	؟	؟	؟	✓	✓
Bashir et al.	✓	✓	×	?	?	؟	؟	✓	✓	✓
Ukpanukpong et al.	✓	✓	✓	?	?	✓	؟	؟	✓	✓
Al-Shaibani et al.	✓	✓	×	?	?	؟	؟	✓	✓	✓

### 3.1 Preparation of garlic extract for therapeutic purposes

The garlic plant has unique medicinal properties that can be used through extract preparation. This extract is rich in bioactive compounds that are responsible for its various therapeutic properties. To prepare garlic extract for therapeutic purposes, fresh and high-quality cloves of garlic should be selected first, and then the outer skin should be removed. Peeled garlic cloves should be crushed or crushed to make them into small pieces. Crushing or crushing garlic helps release its beneficial compounds. Then chopped or crushed garlic is poured into the container and a suitable solvent is added to extract its active compounds. Common solvents for extracting garlic compounds are alcohol or a mixture of alcohol and water. In the next step, the glass container is closed with a tight lid and it is kept in a cool and dark place away from direct sunlight. It is necessary to soak the garlic for a certain period of time, usually 2–4 weeks. During this time, the mixture should be gently stirred every few days to ensure a better extraction process. Afterwards, after the soaking period, the mixture is strained using cheesecloth, a fine mesh strainer, or a coffee filter to remove solid particles. The garlic solids are pressed in the cheese cloth until the juice is completely removed. In the next step, the strained liquid is poured into a dark glass bottle or vial with a closed cap. Light-sensitive compounds in garlic are degraded when exposed to light, so it is important to store the extract in a dark container. It is better to record the date of preparation and the concentration of the extract on the bottle with a label. Then the garlic extract is stored in the refrigerator to maintain its properties and freshness. It can usually be kept for several months, but it is essential that the extract be checked regularly for signs of spoilage and discarded if there is any change in color, smell or texture [58]. In addition, to treat genital problems, garlic bulbs are completely peeled, washed and chopped into small pieces. Then 250 g of chopped garlic is dissolved in distilled water and kept in the refrigerator for 12 h. To prepare the aqueous extract of garlic, 100 g of garlic are homogenized in 250 ml of distilled water. After filtration with Whatman No. 1 filter paper (0.45 μm), 1 ml of this fresh solution per 100 g of body weight, equivalent to 0.4 g of active ingredient per 100 g of body weight, is used orally [59].

### 3.2 Antimicrobial properties

In this review, one of the key results was that garlic has antimicrobial properties that can be effective in fighting various infections that can affect the reproductive system, such as yeast infections and bacterial vaginosis [60]. However, more research is needed to establish the exact mechanism of action and effectiveness of garlic in prevention or treatment through its antimicrobial properties [6]. Garlic has traditionally been valued for its antimicrobial properties and can be effective in healing various infections. Garlic has strong antimicrobial properties against a wide range of bacteria, viruses, fungi and parasites. Garlic may be effective in treating female genital tract infections, including infections caused by *Candida albicans*, a common cause of vaginal yeast infections. Some studies have also shown that garlic may have antimicrobial effects against the bacteria responsible for urinary tract infections [61]. Garlic has been surveyed for its potential role in the prevention and treatment of urinary tract infections (UTIs), which commonly affect women. However, more studies need to be done to confirm this and determine effective doses. In addition, garlic has antifungal properties and can be used in the treatment of fungal infections such as vaginal yeast infections [62]. According to the review and interpretation of the studies included in this review, garlic extract and garlic oil showed inhibitory effects on Candida species. However, careful research should be done to determine its dosage in the treatment of fungal infections. Garlic contains active ingredients *allicin* and *ajoene*, which have strong antimicrobial effects. These compounds are the main factors in the treatment of female genital tract infection, which can be extracted from the garlic plant and used in capsule and ointment forms [63].

### 3.3 Anti-inflammatory properties

This review also found that garlic compounds have anti-inflammatory properties. Inflammation is a common factor in several reproductive disorders such as endometriosis, pelvic inflammatory disease (PID), polycystic ovary syndrome (PCOS), chronic pelvic pain, and ovarian cysts [64]. The anti-inflammatory effects of garlic may be effective in reducing some of the symptoms associated with these disorders, although more specific studies on the reproductive system are needed [65]. Arreola et al. conducted a review study on the immune system modulating and anti-inflammatory effects of garlic compounds. The results obtained from their experimental study showed that garlic modulates the function by stimulating certain types of cells, such as macrophages, lymphocytes, natural killer (NK) cells, dendritic cells and eosinophils with mechanisms such as modulation of cytokine secretion, immunoglobulin production and phagocytosis [17]. Since the dysfunction of the immune system plays an important role in the development and progression of many diseases, several studies were conducted on the use of garlic extract and compounds in the field of immune system modulation. Analysis of the results of these studies showed that the anti-inflammatory activity of garlic is due to the presence of sulfur compounds *allicin* and *ajoene*. Moreover, this review showed that garlic extract modulates cytokine secretion and such modulation can reduce inflammatory manifestations, especially in female reproductive system disorders.

### 3.4 Antioxidant properties

This review showed that garlic extract also has antioxidant activity that may protect against reproductive disorders associated with oxidative stress [66]. In addition to sulfur compounds, garlic also has antioxidant compounds and can increase fertility by reducing lipid peroxidation. However, more research is needed to find out the mechanism of action, dosage and long-term safety considerations [67]. Cherry et al. carried out a study on garlic and fertility. Although their study looked at the effects of garlic on male fertility, it also briefly mentioned the potential benefits of garlic on female fertility. Garlic has antioxidant properties that protect eggs from oxidative stress and improve their quality, potentially increasing fertility [68]. Mostafa et al. conducted a study on the antioxidant effect of garlic (*Allium sativum*) and black seed (*Nigella sativa*) in healthy postmenopausal women. 30 healthy postmenopausal women (mean age 50.31 ± 4.23 years) participated in this study. They took two garlic softgels (each equivalent to 1000 mg of fresh garlic cloves) and raw ground black seed powder at a dose of 3 g per day for 8 weeks. In their study, the oxidizing activity of malondialdehyde in plasma and the activity of antioxidants superoxide dismutase and glutathione peroxidase in red blood cells were investigated [69]. The results of their study showed that significant low levels of plasma malondialdehyde were observed along with increased activity of glutathione peroxidase and superoxide dismutase. From their study, it was concluded that menopause is related to the increase of oxidative stress and the decrease of some antioxidant parameters. The consumption of raw garlic and its extract may have a positive effect on improving the balance between blood oxidants and antioxidants in healthy postmenopausal women [69]. This review showed that the antioxidant properties of garlic may protect ovum and reproductive organs from oxidative damage and reduce the risk of infertility [70]. Asadpour et al. reported that garlic has antioxidant activity and prevents the activity of oxygen peroxide due to the presence of vitamin E in garlic extract [71]. Garlic compounds such as *allicin* and *ajoene* have antioxidant properties. These antioxidants can protect cells from oxidative damage and potentially reduce the risk of reproductive disorders. In addition, reactive oxygen species present in garlic extract play a very important role in intracellular signaling processes. Reactive oxygen species play a significant role in infertility due to excessive production of oxidants and thus reducing the ability of the reproductive system's antioxidant system [72].

### 3.5 Hormonal regulation

The results of included studies showed that garlic compounds have potential effects on menstrual cycle regulation, hormonal regulation and fertility. However, some studies also reported adverse effects, such as changes in endometrial thickness and hormonal changes [25,73]. This review showed that garlic plays a role in reducing menstrual pain and improving menstrual regularity. Garlic has analgesic properties that are effective in relieving menstrual pain. In addition, evidence from the reviewed studies showed that regular consumption of garlic was correlated with menstrual cycle regularity, indicating garlic's potential role in hormonal regulation. Based on an analysis of studies, garlic may have modulating effects on estrogen levels in the body, which could potentially affect the female reproductive system [74,75]. Some studies have shown that certain compounds in garlic have estrogen-like properties and help regulate hormone levels. Some studies also report that garlic may play a role in modulating hormone levels, especially estrogen. Garlic can be effective in regulating menstrual cycles or reducing the symptoms of diseases such as polycystic ovary syndrome (PCOS) [76]. However, the evidence is limited and more laboratory studies are needed to draw firm conclusions. Several studies have shown that garlic may have a positive effect on the regulation of female hormones. Examining the effects of garlic extract on the reproductive potential of female rats by Nazifi et al. demonstrated that the administration of garlic extract for 14 days significantly changed the serum levels of estradiol and progesterone to normal levels, which indicates the potential positive effect of garlic on the secretion and Regulation of hormones. Garlic supplementation has been shown to reduce estrogen, progesterone, and luteinizing hormone (LH) levels, thereby reducing the hormonal imbalances that commonly occur in PCOS. Garlic has been found to contain compounds such as *allicin*, which may help regulate estrogen levels [73,77,78]. Modaresi et al. reported that garlic can increase the secretion of estrogen and progesterone blood levels in women. This action occurs with the formation of more follicles [79,80]. Hammami et al. concluded that garlic contains phytoestrogens that have a direct effect on estrogen levels [81]. Additionally, Oi et al.'s results showed that garlic supplementation increases LH from the pituitary gland, which stimulates the release of estrogen and progesterone [82]. Ahmadian et al. investigated the effect of garlic tablets on protein oxidation biomarkers in postmenopausal women with osteoporosis. This study was a double-blind randomized clinical trial that was conducted on 42 postmenopausal women in Yazd during 2013–2014. Women with osteoporosis were randomly assigned to two garlic groups (GG) and placebo group (PG). GG participants took two garlic tablets daily for 1 month, and PG participants took placebo tablets in the same manner. After 30 days, plasma levels of carbonyl groups (PCO), total antioxidant capacity (TAC) and advanced oxidation protein products (AOPPs) were analyzed by spectrophotometric assay. The results of their study showed that garlic tablets reduced PCO plasma levels. According to the results obtained from their study, the parameters of the placebo group before and after the study did not show any significant difference. Moreover, the level of MDA before taking the drug was also reduced compared to before in the garlic group [83]. This study indicated that garlic tablets had decreased PCO plasma levels (47.37 ± 5.98 vs. 19.62 ± 3.40 nM, p ≤ 0.001, before and after the study, respectively), AOPPs (738.95 ± 151.86 vs. 585.12 ± 209.99 μM, p ≤ 0.008, before and after the study, respectively), and increased TAC (11.34 ± 10.80 vs. 47.93 ± 17.80, p ≤ 0.001, before and after the study, respectively). The levels of MDA before taking the drug in comparison to before Garlic group was also reduced (1.30 ± 1.04 vs. 0.92 ± 0.81 μM, p = 0.01, before and after the study, respectively) [83]. In another study Jafari et al. surveyed the effect of garlic supplementation on premenstrual disorders. The results of their study showed that there was no statistically significant difference between the two groups of premenstrual symptoms before the intervention. After treatment with garlic for three consecutive cycles, the total score of the severity of premenstrual symptoms significantly reduced from 34.09 ± 7.31 to 11.21 ± 7.17. In the placebo group, this score changed from 33.35 ± 7.96 to 24.28 ± 7.22. The difference between mean changes in the two groups was 13.78, with a 95 % Confidence Interval (CI) of 11.23–16.33. Besides, no serious side effects were observed in these two study groups. The results of their study showed that the consumption of garlic can be considered as a complementary treatment in the prevention and treatment of premenstrual disorders [84]. Additionally, this review identified and reviewed some studies that reported compounds in garlic may have contraceptive properties. However, these findings are limited and more research is needed to confirm such claims. According to the selected studies, the effects of garlic on breastfeeding have not been well studied. There is no conclusive evidence that garlic has a negative effect on breastfeeding. Although some studies have reported that garlic may change the taste or smell of breast milk, potentially affecting infant feeding behavior.

### 3.6 Fertility and reproductive health

This review found that garlic may have positive effects on women's fertility and reproductive health. Garlic contains antioxidants such as selenium and vitamin C, which may protect the ovum from oxidative damage. Shah et al. investigated the capacity of diallyl trisulfide (DAT) to reduce the fertility of *Sitotroga cerealella*. The results of their study showed that DAT negatively regulated the fertility of *S. cerealella* [85]. Raji et al. evaluated the effect of garlic extract on some aspects of reproduction in female albino rats (Wistar strain). In their study, there was a significant increase in the length of the right uterine horn and a decrease in the length of the left uterine horn (P < 0.05) in groups B, C, D and E compared to group A. In addition, a significant difference (p > 0.05) was reported in pregnant women without abortion in infant size, live birth weight and infant mortality among the groups. These results showed that the aqueous extract of garlic had no harmful effect on the reproductive performance of female mice [33]. A review of several studies showed that garlic has a beneficial effect on ovarian activity and plays an important role in improving women's reproductive outcomes. Garlic affects the hypothalamus-pituitary function due to its diallyl disulfide compound. Garlic can also increase fertility possibly due to its antioxidant properties. However, clinical trials are needed to investigate these mechanisms in detail [86].

### 3.7 Menstruation and menstrual pain

In this review, it was found that garlic has cardiovascular benefits, including lowering cholesterol levels and lowering blood pressure. These effects may indirectly benefit the female reproductive system with better blood flow in the pelvic area, which is essential for healthy menstrual cycles and overall reproductive health. Faroughi et al. studied the effects of garlic extract on the symptoms of premenstrual syndrome (PMS) in a double-blind randomized clinical trial with placebo. The results of their study showed that garlic supplementation significantly reduced the severity of PMS symptoms. They concluded from this study that garlic may have positive effects on menstrual disorders and mood swings in women [87]. By affecting the anterior pituitary gland, diallyl disulfide stimulates basophil cells and the secretion of sex hormones LH. It is important to note that the effects of garlic on the female reproductive system and hormonal regulation are still being investigated and more research is needed to develop a clear understanding of these effects. While garlic can be a healthy addition to a balanced diet, it's always best to consult your doctor before making any significant changes to your diet because the compound *allicin* may have vasodilating effects [88]. This feature can dilate blood vessels and improve blood flow. Garlic has traditionally been used to help regulate menstruation and reduce menstrual pain. The herb is believed to have properties that can stimulate the uterus and promote blood flow, which may help regulate irregular periods. However, scientific evidence to support these claims is limited and more research is needed to confirm these effects.

### 3.8 Anticancer properties

A review of these studies showed that garlic may have anticancer effects in certain types of cancer, including breast, ovarian, and cervical cancer. Garlic contains compounds such as *allicin*, which have been shown to inhibit the growth of cancer cells, reduce tumor size, and induce apoptosis in cancer cells. However, more research is needed to determine the specific effects of garlic on different types of cancer [89]. Garlic is rich in antioxidants and anti-inflammatory compounds that may have positive effects on overall health, including the female reproductive system. Chronic inflammation and oxidative stress can cause the development of cancer [90]. By reducing inflammation and oxidative stress, garlic may help protect cells from damage. In several studies, the ability of garlic extract to inhibit the growth and progression of bladder cancer has been mentioned [91]. Garlic's ability to inhibit cancer growth is based on preventing tumor angiogenesis and stimulating helper T cells. Garlic has a positive effect on the immune system and reduces the progress of cancer. According to a review of these studies, garlic has shown strong anticancer activity, especially in relation to tumors of the digestive tract [92]. Furthermore, according to these included studies, garlic consumption reduces the risk of esophageal, stomach, and colon cancer. Several bioactive compounds in garlic, including DATS, *allicin*, DADS, diallyl sulfide, and allyl mercaptan, have anticancer properties [93]. It is important to note that although garlic is promising in some areas, it is not a definitive cure for cancer. There are limited studies on the specific effects of garlic on female genital cancers [94]. However, due to its potential anticancer properties, garlic can have a favorable effect on the prevention and progression of cancers of the female reproductive system.

### 3.9 Garlic effects in Persian medicine (PM)

Garlic is known as a valuable medicinal plant in Persian medicine (PM) with various health benefits. For centuries, this plant has been used in traditional Iranian medicine to treat various diseases. Today, people are more inclined to use complementary and alternative medicine (CAM) for premenstrual disorder (PMD) due to side effects and drug interactions [95]. PM as a CAM method suggests appropriate approaches. Among the various CAM methods, the use of herbal medicines is the most popular method. According to sources of Iranian medicine, garlic is suggested as one of the herbal medicines that can be effective in PMS by reducing blood viscosity and regulating menstruation [84]. The mechanism of garlic's effectiveness is not fully understood, but a number of studies have reported that garlic dilutes the blood and reduces blood clotting time [96]. Therefore, it can facilitate the menstrual process. Various PM-related studies have investigated the various uses of garlic in fertility-related problems, including menstrual regulation, prevention, and treatment of menstrual retention [97]. In these studies, the various uses of garlic in the treatment of infertility as well as the prevention and treatment of histrionic diseases have been mentioned [98]. Therefore, if there is sufficient clinical evidence, garlic can be prescribed as a medicinal supplement in the prevention and treatment of female reproductive diseases along with drugs [84].

### 3.10 Strengths and weaknesses of the systematic review

#### 3.10.1 Strengths

This systematic review followed a robust research methodology, ensuring that no relevant studies were overlooked. This method helped minimize bias and provided a comprehensive understanding of the subject. In addition, this review included a wide range of animal experiments as well as laboratory experiments. This increased accuracy and specificity in this field. The review specified clear inclusion and exclusion criteria, and only studies that met certain quality standards were included. This strengthened the validity of the survey results. The review also critically assessed the quality of the included studies, assessing factors such as study design, sample size, and statistical analysis. This evaluation increased the validity of the findings and results of the review. Finally, this review provided a summary of the main findings of the included studies. It helps readers quickly understand the key concepts and implications of garlic's therapeutic effects on the female reproductive system.

#### 3.10.2 Weaknesses

This review included studies with different methodologies, which made comparison and synthesis of results challenging. This heterogeneity may have weakened the strength of the review's overall conclusions. Besides, a number of studies included in this review focused on short-term effects, which may limit the review's ability to assess the long-term therapeutic benefits of garlic. Long-term studies will provide a more comprehensive understanding of the sustainability and potential risks associated with the consumption of garlic.

#### 3.10.3 Future perspective

Despite the promising results, the available literature on the therapeutic effects of garlic on the female reproductive system is limited and heterogeneous in terms of study design, sample size, and methodology. More research is needed to provide stronger evidence and establish mechanisms of action for the beneficial effects of garlic. Future studies should consider randomized controlled trials with larger sample sizes and long-term follow-ups to evaluate the efficacy, safety, and possible side effects of garlic supplementation. In addition, investigating the active components of garlic and its specific action mechanisms will help to better understand its therapeutic effects.

## 4 Conclusions

Garlic plant (*Allium sativum*) has been considered for centuries because of its taste characteristics and therapeutic effects. In recent years, the therapeutic effects of garlic on the function and disorders of the female reproductive system have been discussed. According to this systematic review, garlic had several therapeutic effects on the female reproductive system. Garlic has been found to have antimicrobial activity against various pathogens such as *Candida albicans* that affect the female reproductive system. Thus, the plant may be effective in preventing or treating infections, including vaginal yeast infections and urinary tract infections. Garlic has anti-inflammatory properties that may reduce inflammation in the reproductive system. This can be effective for women with diseases such as pelvic inflammatory disease or endometriosis, where inflammation plays an important role. Some studies have demonstrated that garlic may affect hormonal balance. Additionally, garlic has been found to regulate estrogen levels and can potentially improve conditions associated with hormonal imbalances, such as polycystic ovary syndrome (PCOS). Garlic contains various antioxidants that can protect cells from damage caused by oxidative stress. This antioxidant activity has positive effects on the reproductive system by reducing oxidative damage. Furthermore, garlic consumption can reduce menopausal symptoms such as hot flashes and mood swings. More research is needed to confirm this correlation and determine the mechanisms of these effects. Although some animal studies have shown that garlic may increase fertility, there is currently limited evidence of its direct effect on female fertility. Overall, the reviewed studies showed that garlic may have therapeutic effects on the female reproductive system. Garlic shows antimicrobial, anti-inflammatory, hormonal balance and antioxidant properties that can promote reproductive health. However, further research, especially in human trials, is needed to confirm these findings and determine optimal dosage and administration methods.

## Ethics statement

Not applicable.

## Funding statement

This research did not receive any specific grant from funding agencies in the public, commercial, or not-for-profit sectors.

## Additional information

No additional information is available for this paper.

## Data availability statement

The data associated with the study was not deposited into a publicly available repository because no data was used for the research described in the article.

## CRediT authorship contribution statement

**Fatemeh Jafari:** Methodology, Conceptualization. **Somayyeh Khalilzadeh:** Supervision. **Fatemeh Nejatbakhsh:** Writing – review & editing, Maziar Naderi, Writing – review & editing.

## Declaration of competing interest

The authors declare that they have no known competing financial interests or personal relationships that could have appeared to influence the work reported in this paper.

## Figures and Tables

**Table:** 

Section and Topic	Item #	Checklist item	Reported (Yes/No)
TITLE			Yes
Title	1	Identify the report as a systematic review.	**Yes**
**ABSTRACT**			**Yes**
Abstract	2	See the PRISMA 2020 for Abstracts checklist.	**Yes**
**INTRODUCTION**			**Yes**
Rationale	3	Describe the rationale for the review in the context of existing knowledge.	**Yes**
Objectives	4	Provide an explicit statement of the objective(s) or question(s) the review addresses.	**Yes**
**METHODS**			**Yes**
Eligibility criteria	5	Specify the inclusion and exclusion criteria for the review and how studies were grouped for the syntheses.	**Yes**
Information sources	6	Specify all databases, registers, websites, organizations, reference lists and other sources searched or consulted to identify studies. Specify the date when each source was last searched or consulted.	**Yes**
Search strategy	7	Present the full search strategies for all databases, registers and websites, including any filters and limits used	**Yes**
Selection process	8	Specify the methods used to decide whether a study met the inclusion criteria of the review, including how many reviewers screened each record and each report retrieved, whether they worked independently, and if applicable, details of automation tools used in the process.	**Yes**
Data collection process	9	Specify the methods used to collect data from reports, including how many reviewers collected data from each report, whether they worked independently, any processes for obtaining or confirming data from study investigators, and if applicable, details of automation tools used in the process.	**Yes**
Data items	10 a	List and define all outcomes for which data were sought. Specify whether all results that were compatible with each outcome domain in each study were sought (e.g. for all measures, time points, analyses), and if not, the methods used to decide which results to collect.	**Yes**
	10 b	List and define all other variables for which data were sought (e.g. participant and intervention characteristics, funding sources). Describe any assumptions made about any missing or unclear information.	**Yes**
Study risk of bias assessment	11	Specify the methods used to assess risk of bias in the included studies, including details of the tool(s) used, how many reviewers assessed each study and whether they worked independently, and if applicable, details of automation tools used in the process.	**Yes**
Effect measures	12	Specify for each outcome the effect measure(s) (e.g. risk ratio, mean difference) used in the synthesis or presentation of results.	**Yes**
Synthesis methods	13a	Describe the processes used to decide which studies were eligible for each synthesis (e.g. tabulating the study intervention characteristics and comparing against the planned groups for each synthesis (item #5)).	**Yes**
	13 b	Describe any methods required to prepare the data for presentation or synthesis, such as handling of missing summary statistics, or data conversions	**Yes**
	13 c	Describe any methods used to tabulate or visually display results of individual studies and syntheses.	**Yes**
	13 d	Describe any methods used to synthesize results and provide a rationale for the choice(s). If meta-analysis was performed, describe the model(s), method(s) to identify the presence and extent of statistical heterogeneity, and software package(s) used.	**Yes**
	13e	Describe any methods used to explore possible causes of heterogeneity among study results (e.g. subgroup analysis, meta-regression).	**Yes**
	13f	Describe any sensitivity analyses conducted to assess robustness of the synthesized results.	**Yes**
Reporting bias assessment	14	Describe any methods used to assess risk of bias due to missing results in a synthesis (arising from reporting biases).	**Yes**
Certainty assessment	15	Describe any methods used to assess certainty (or confidence) in the body of evidence for an outcome.	**Yes**
Study selection	16a	Describe the results of the search and selection process, from the number of records identified in the search to the number of studies included in the review, ideally using a flow diagram.	**Yes**
	16 b	Cite studies that might appear to meet the inclusion criteria, but which were excluded, and explain why they were excluded.	**Yes**
Study characteristics	17	Cite each included study and present its characteristics.	**Yes**
Risk of bias in studies	18	Present assessments of risk of bias for each included study.	**Yes**
Results of individual studies	19	For all outcomes, present, for each study: (a) summary statistics for each group (where appropriate) and (b) an effect estimate and its precision (e.g. confidence/credible interval), ideally using structured tables or plots.	**Yes**
Results of syntheses	20a	For each synthesis, briefly summaries the characteristics and risk of bias among contributing studies.	**Yes**
	20 b	Present results of all statistical syntheses conducted. If meta-analysis was done, present for each the summary estimate and its precision (e.g. confidence/credible interval) and measures of statistical heterogeneity. If comparing groups, describe the direction of the effect.	**Yes**
	20c	Present results of all investigations of possible causes of heterogeneity among study results.	**Yes**
	20 d	Present results of all sensitivity analyses conducted to assess the robustness of the synthesized results.	**Yes**
Reporting biases	21	Present assessments of risk of bias due to missing results (arising from reporting biases) for each synthesis assessed.	**Yes**
Certainty of evidence	22	Present assessments of certainty (or confidence) in the body of evidence for each outcome assessed.	**Yes**
**DISCUSSION**			**Yes**
Discussion	23a	Provide a general interpretation of the results in the context of other evidence.	**Yes**
	23 b	Discuss any limitations of the evidence included in the review.	**Yes**
	23c	Discuss any limitations of the review processes used.	**Yes**
	23 d	Discuss implications of the results for practice, policy, and future research.	**Yes**
**OTHER INFORMATION**			**Yes**
Registration and protocol	24a	Provide registration information for the review, including register name and registration number, or state that the review was not registered.	**Yes**
	24 b	Indicate where the review protocol can be accessed, or state that a protocol was not prepared.	**Yes**
	24c	Describe and explain any amendments to information provided at registration or in the protocol.	**Yes**
Support	25	Describe sources of financial or non-financial support for the review, and the role of the funders or sponsors in the review.	**Yes**
Competing interests	26	Declare any competing interests of review authors.	**Yes**
Availability of data, code and other materials	27	Report which of the following are publicly available and where they can be found: template data collection forms; data extracted from included studies; data used for all analyses; analytic code; any other materials used in the review.	**Yes**

From: Page MJ, McKenzie JE, Bossuyt PM, Boutron I, Hoffmann TC, Mulrow CD et al. The PRISMA 2020 statement: an updated guideline for reporting systematic reviews. BMJ 2021; 372:n71. https://doi.org/10.1136/bmj.n71.

**Table:** 

Section and Topic	Item #	Checklist item	Reported (Yes/No)
TITLE			Yes
Title	1	Identify the report as a systematic review.	**Yes**
**BACKGROUND**			**Yes**
Objectives	2	Provide an explicit statement of the main objective(s) or question(s) the review addresses.	**Yes**
**METHODS**			**Yes**
Eligibility criteria	3	Specify the inclusion and exclusion criteria for the review.	**Yes**
Information sources	4	Specify the information sources (e.g. databases, registers) used to identify studies and the date when each was last searched.	**Yes**
Risk of bias	5	Specify the methods used to assess risk of bias in the included studies.	**Yes**
Synthesis of results	6	Specify the methods used to present and synthesize results.	**Yes**
**RESULTS**			**Yes**
Included studies	7	Give the total number of included studies and participants and summaries relevant characteristics of studies.	**Yes**
Synthesis of results	8	Present results for main outcomes, preferably indicating the number of included studies and participants for each. If meta-analysis was done, report the summary estimate and confidence/credible interval. If comparing groups, indicate the direction of the effect (i.e. which group is favored)	**Yes**
**DISCUSSION**			**Yes**
Limitations of evidence	9	Provide a brief summary of the limitations of the evidence included in the review (e.g. study risk of bias, inconsistency and imprecision).	**Yes**
Interpretation	10	Provide a general interpretation of the results and important implications	**Yes**
**OTHER**			**Yes**
Funding	11	Specify the primary source of funding for the review.	**Yes**
Registration	12	Provide the register name and registration number.	**Yes**

From: Page MJ, McKenzie JE, Bossuyt PM, Boutron I, Hoffmann TC, Mulrow CD et al. The PRISMA 2020 statement: an updated guideline for reporting systematic.

Reviews: BMJ 2021; 372:n71. https://doi.org/10.1136/bmj.n71.

## References

[1] Bulavenko O. (2021). Problems and challenges to women’s reproductive health in the 21th century.

[2] Tozun M. (2022). Knowledge, attitudes, and opinions of health professionals and students on traditional and complementary medicine practices in Turkey: a systematic review and meta-analysis. European J. Environ. Public Health.

[3] Naderi M. (2022). Effect of ozone on the inactivation of indoor airborne viruses with the COVID-19 virus approach: a systematic review. Tehran Univ. Medical J. TUMS Publicat..

[4] Ansary J. (2020). Potential health benefit of garlic based on human intervention studies: a brief overview. Antioxidants.

[5] Rivlin R.S. (2001). Historical perspective on the use of garlic. J. Nutr..

[6] Tesfaye A. (2021). Revealing the therapeutic uses of garlic (allium sativum) and its potential for drug discovery. Sci. World J..

[7] Mathew B., Biju R. (2008). Neuroprotective effects of garlic a review. Libyan J. Med..

[8] Chidinma O. (2019). Therapeutic effects of garlic: a review. Sci. J. Biol. & Life Sci..

[9] Ozioma E O.J., Chinwe O.A.N. (2019). Herbal medicines in African traditional medicine. Herbal Med..

[10] Kunle (2012). Standardization of herbal medicines-A review. Int. J. Biodiv. Conservat..

[11] Ebrahimnejad M. (2022). Complicated role of exercise in modulating memory: a discussion of the mechanisms involved. Neurochem. Res..

[12] Jiao M. (2022). Comparison of herbal medicines used for women’s menstruation diseases in different areas of the world. Front. Pharmacol..

[13] Suleria H.A.R. (2015). Garlic (Allium sativum): diet based therapy of 21st century–a review. Asian Pacific J. Trop. Disease.

[14] Verma T. (2023). Medicinal and therapeutic properties of garlic, garlic essential oil, and garlic-based snack food: an updated review. Front. Nutr..

[15] Zugaro S., Benedetti E., Caioni G. (2023). Garlic (Allium sativum L.) as an Ally in the Treatment of inflammatory bowel diseases. Current Issues Molecular Biol..

[16] Adaki S. (2014). *Garlic: Review of literature.* Indian journal of cancer.

[17] Arreola R. (2015). Immunomodulation and anti-inflammatory effects of garlic compounds. J. Immunol. Res..

[18] Kimura S. (2017). Black garlic: a critical review of its production, bioactivity, and application. J. Food Drug Analy..

[19] Esmaeili M., Abedian Kenari A., Rombenso A. (2017). Effects of fish meal replacement with meat and bone meal using garlic (Allium sativum) powder on growth, feeding, digestive enzymes and apparent digestibility of nutrients and fatty acids in juvenile rainbow trout (Oncorhynchus mykiss Walbaum, 1792). Aquacult. Nutr..

[20] Elosta A., Ghous T., Ahmed N. (2012). Natural products as anti-glycation agents: possible therapeutic potential for diabetic complications. Curr. Diabetes Rev..

[21] Yarbrough V.L., Winkle S., Herbst-Kralovetz M.M. (2015). Antimicrobial peptides in the female reproductive tract: a critical component of the mucosal immune barrier with physiological and clinical implications. Hum. Reprod. Update.

[22] Walke G. (2023). The impact of oxidative stress on male reproductive function: exploring the role of antioxidant supplementation. Cureus.

[23] Sumpter C., Torondel B. (2013). A systematic review of the health and social effects of menstrual hygiene management. PLoS One.

[24] Singh R., Singh K. (2019). Garlic: a spice with wide medicinal actions. J. Pharmacognosy Phytochemist..

[25] Falahatian S., Haddad R., Pakravan N. (2022). Modulatory effects of R10 fraction of garlic (Allium sativum L.) on hormonal levels, T cell polarization, and fertility-related genes in mice model of polycystic ovarian syndrome. J. Ovarian Res..

[26] Lorand T., Vigh E., Garai J. (2010). Hormonal action of plant derived and anthropogenic non-steroidal estrogenic compounds: phytoestrogens and xenoestrogens. Curr. Med. Chem..

[27] Martin-Hidalgo D. (2019). Antioxidants and male fertility: from molecular studies to clinical evidence. Antioxidants.

[28] Gholiof M., Luca A.-D., Wessels J.M. (2022). The female reproductive tract microbiotas, inflammation, and gynecological conditions. Front. Reproduct. Health.

[29] Musavi H. (2018). Effect of garlic (Allium sativum) on male fertility: a systematic review. J. Herbmed Pharmacol..

[30] Abedini R. (2021). Determination of melamine contamination in chocolates containing powdered milk by high-performance liquid chromatography (HPLC). J. Environm. Health Sci. Eng..

[31] Gholami S., Naderi M., Moghaddam A.M. (2018). Investigation of the survival of bacteria under the influence of supporting electrolytes KCl, CuI and NaBr in the electrochemical method. Ment. Health.

[32] Ozma M.A. (2022). A critical review on the nutritional and medicinal profiles of garlic’s (Allium sativum L.) bioactive compounds. Food Rev. Int..

[33] Raji L. (2012). The effects of aqueous extract of allium sativum (garlic) on some aspects of reproduction in the female albino rat (wistar strain). Global Vet..

[34] Waseem N., Rehman S. (2015). The efficiency of garlic extract in prevention of lead acetate toxicity on fallopian tube–a hormonal study. Biol. Med..

[35] Bashir Y., Tahir M., Lone K. (2017). Effect of aqueous garlic extract on androgen induced changes in ovaries of prepubertal female albino rats. Biomedica.

[36] Page M.J. (2021). PRISMA 2020 explanation and elaboration: updated guidance and exemplars for reporting systematic reviews. BMJ.

[37] Hooijmans C.R. (2014). SYRCLE’s risk of bias tool for animal studies. BMC Med. Res. Methodol..

[38] Valente C., Aboua G., Du Plessis S.S. (2014).

[39] El-Saber Batiha G. (2020). Chemical constituents and pharmacological activities of garlic (Allium sativum L.): a review. Nutrients.

[40] Chang M.-M. (2019). Effect of diallyl trisulfide on the reproductive behavior of the grain moth, Sitotroga cerealella (Lepidoptera: gelechiidae). Insects.

[41] Thuy B.T.P. (2020). Investigation into SARS-CoV-2 resistance of compounds in garlic essential oil. ACS Omega.

[42] Krauth D., Woodruff T.J., Bero L. (2013). Instruments for assessing risk of bias and other methodological criteria of published animal studies: a systematic review. Environ. Health Perspect..

[43] Ghyasi R., Moslehi A., Naderi R. (2019). Combination effect of voluntary exercise and Garlic (Allium sativum) on oxidative stress biomarkers and lipid profile in healthy rats. Pharmaceut. Sci..

[44] Desai K. (2015). Protective efficacy of Allium sativum on deltamethrin induced toxicity in reproductive tissues of male mice. Int. J. Pharmaceut. Sci. Res..

[45] Elkelawy H. (2020). Effect of garlic (Allium sativum) on hematological, biochemical, hormonal and fertility parameters of male bouscat rabbits. Egyptian J. Rabbit Sci..

[46] Hajiuon B. (2014). Effects of garlic (Allium sativum L.) hydroalcoholic extract on estrogen, progesterone and testosterone levels in rats exposed to cell phone radiation. Zahedan J. Res. Med. Sci..

[47] Jafari S., Farokhi F., Sadeghi A. (2021). Comparative effects of garlic (Allium sativum) powder and atorvastatin in female reproductive system of hypercholesterolemic rats: a histological and biochemical evaluation. J. Shahrekord Univ. Med. Sci..

[48] Risikat K.E. (2022). Comparative oestrogenic effects of Allium sativum and Allium cepa in ovariectomised rats. Anatomy J. Africa.

[49] Batool S. (2022). Curative potentials of garlic (allium sativum) extract against di-(2-ethylhexyl) phthalate induced reproductive toxicity in female mice: amelioration of DEHP-induced toxicity by garlic extract. Proc. Pakistan Acad. Sci.: B. Life Environ. Sci..

[50] Iram F. (2022). Effect of aqueous garlic (Allium sativum) extract against di-(2-ethylhexyl) phthalate induced reproductive toxicity in male mice. Andrologia.

[51] Hagag O.Y.A.-E. (2023). Effect of feeding pomegranate (punica granatum) peel and garlic (allium sativum) on antioxidant status and reproductive efficiency of female rabbits. Veterinary Sci..

[52] Parvez M. (2015). Antifertility activity of methanol bulb extract of Allium sativum on Swiss albino male mice and teratogenic effect on neonates of female mice. Global J. Pharmacol..

[53] Kadir R. (2018). Oestrogenic effects of onion and garlic extracts: potential alternatives to synthetic oestradiol?. Trop. J. Health Sci..

[54] Ene, C.B., et al., The Effect of Allium Sativum (Garlic) on Pregnancy, Fetal Weights, and Some Hematological Parameters in Albino Rats..

[55] Sheweita S.A. (2022). Antioxidants (selenium and garlic) alleviated the adverse effects of tramadol on the reproductive system and oxidative stress markers in male rabbits. Sci. Rep..

[56] Ukpanukpong, R., et al., Hormonal and Electrolyte Assessment on the Effect of Garlic (Allium Sativum), Vitamin C and E in Tramadol Induced Toxicity in Female Wistar Rats..

[57] Al-Shaibani, S.W., W.H. AL-hashimhi, and H.S. Bshibash, Histological Study of Aqueous Extracts Leek Allium porrum L. In Female Reproductive System of Laboratory White Rats..

[58] Singh P. (2014). Shelf life evaluation of raw chicken meat emulsion incorporated with clove powder, ginger and garlic paste as natural preservatives at refrigerated storage (4±1° C). Int. Food Res. J..

[59] Kallel F. (2015). Polysaccharide from garlic straw: extraction, structural data, biological properties and application to beef meat preservation. RSC Adv..

[60] Felix T.C. (2019). D.V.D. de Brito Röder, and R. dos Santos Pedroso, Alternative and complementary therapies for vulvovaginal candidiasis. Folia Microbiol..

[61] Bhatwalkar S.B. (2021). Antibacterial properties of organosulfur compounds of garlic (Allium sativum). Front. Microbiol..

[62] Poulios E. (2021). Medicinal plants consumption against urinary tract infections: a narrative review of the current evidence. Expert Rev. Anti-infect. Ther..

[63] Riesute R. (2021). Effect of yeasts on food quality and safety and possibilities of their inhibition. Trends Food Sci. Technol..

[64] Peter K., Singh B., Kumar P. (2021). Zero hidden hunger: role of vegetables. Vegetable Science.

[65] Karthikraja S. (2016). Pre-clinical study of Herbo Mineral Formulation Rasa Karpoora Kuligai for its anti-cancer, anti-oxidant and anti-inflammatory activities, Government Siddha Medical College, Palayamkottai.

[66] Colín-González A.L. (2012).

[67] Donma M.M., Donma O. (2020). The effects of allium sativum on immunity within the scope of COVID-19 infection. Med. Hypotheses.

[68] Cherry R. (2014).

[69] Mostafa R.M. (2013). Antioxidant effect of garlic (Allium sativum) and black seeds (Nigella sativa) in healthy postmenopausal women. SAGE Open Med..

[70] Dorrigiv M., Zareiyan A., Hosseinzadeh H. (2020). Garlic (Allium sativum) as an antidote or a protective agent against natural or chemical toxicities: a comprehensive update review. Phytother Res..

[71] Asadpour R. (2013). Veterinary Research Forum: an International Quarterly Journal.

[72] Nadeem M.S. (2021). Allicin, an antioxidant and neuroprotective agent, ameliorates cognitive impairment. Antioxidants.

[73] Noh S. (2020). Role of antioxidant natural products in management of infertility: a review of their medicinal potential. Antioxidants.

[74] McEwen B.J. (2015). The influence of herbal medicine on platelet function and coagulation: a narrative review. Semin. Thromb. Hemost. Thieme Medical Publishers.

[75] Ciebiera M. (2021). Nutrition in gynecological diseases: current perspectives. Nutrients.

[76] Sharma N. (2023). A review on the pharmacological potential of Indian spices in polycystic ovarian syndrome. J. Reproduct. Healthcare Med..

[77] Raeeszadeh M., Saleh Hosseini S.M., Amiri A.A. (2022). Impact of co-administration of N-acetylcysteine and vitamin E on cyclophosphamide-induced ovarian toxicity in female rats. J. Toxicol..

[78] Sulekha S.M.P. (2022). Traditional siddha approach to treatment of cognitive impairment in menopause. Current Pharmacol. Reports.

[79] Modaresi M., Taromsari S. (2013). Effect of garlic hydro-alcoholic extract on pituitary-gonad axis in female mice.

[80] Nurhayati N. (2023). Effect of using black garlic instead of fresh garlic in the ration on the performances and blood cholesterol properties of quail. J. Agripet.

[81] Hammami I. (2013). Effects of garlic fractions consumption on male reproductive functions. J. Med. Food.

[82] Oi Y. (2001). Garlic supplementation increases testicular testosterone and decreases plasma corticosterone in rats fed a high protein diet. J. Nutr..

[83] Ahmadian F. (2017). The effect of consumption of garlic tablet on proteins oxidation biomarkers in postmenopausal osteoporotic women: a randomized clinical trial. Electron. Physician.

[84] Jafari F. (2021). Effect of garlic (Allium sativum) supplementation on premenstrual disorders: a randomized, double-blind, placebo-controlled trial. Evid. base Compl. Alternative Med..

[85] Shah S. (2023). Diallyl trisulfide, a Biologically active component of garlic essential oil, decreases male fertility in Sitotroga cerealella by impairing dimorphic spermatogenesis, sperm motility and lipid homeostasis. Cells.

[86] Li B. (2023). Evaluation of expression of Cytochrome P450 aromatase and inflammatory, oxidative, and apoptotic markers in testicular tissue of obese rats (Pre) treated with garlic powder. Evid. base Compl. Alternative Med..

[87] Faroughi F. (2018). Effects of garlic pill on blood glucose level in borderline gestational diabetes mellitus: a randomized controlled trial. Iran. Red Crescent Med. J..

[88] Kochhar K. (2017). The role of traditional diet and yoga for infertility: a blend and balance of traditional knowledge and modern medicine.

[89] Abedini R. (2023). Investigation of melamine and cyanuric acid concentration in several brands of liquid milk and its non-carcinogenic risk assessment in adults and infants. J. Food Sci. Technol..

[90] Huang C. (2023). Non-coding RNAs/DNMT3B axis in human cancers: from pathogenesis to clinical significance. J. Transl. Med..

[91] Shin S.-S. (2017). HSPA6 augments garlic extract-induced inhibition of proliferation, migration, and invasion of bladder cancer EJ cells; Implication for cell cycle dysregulation, signaling pathway alteration, and transcription factor-associated MMP-9 regulation. PLoS One.

[92] Kim W.T. (2018). The anticancer effects of garlic extracts on bladder cancer compared to cisplatin: a common mechanism of action via centromere protein M. Am. J. Chin. Med..

[93] Wang Y. (2022). Association and mechanism of garlic consumption with gastrointestinal cancer risk: a systematic review and meta-analysis. Oncol. Lett..

[94] Imaizumi V.M. (2022). Garlic: a systematic review of the effects on cardiovascular diseases. Crit. Rev. Food Sci. Nutr..

[95] Timba P.P., Giri S.G., Panchal R.V. (2019). Health benefits and possible risks of turmeric, garlic and ginger: a short. Health.

[96] Khorasani M.A. (2001).

[97] Qarshi I. (2008).

[98] Avicenna H., Al-Qanon fi al-Tibb (Canon on medicine), vol. 2. Beirut Lebanon: Alalami Library Publication 21 (7) (2005) 9-21.

